# Efficient modification of B-lynch brace suture for management of postpartum hemorrhage in developing countries: a report of two cases

**DOI:** 10.1093/jscr/rjaf545

**Published:** 2025-07-18

**Authors:** Sami Jomaa, Martha Alhajjeh, Walaa Alhasan, Lutfia Alrakik, Dema Adwan

**Affiliations:** General Surgery Department, Damascus Hospital, Damascus, Syria; University Hospital of Obstetrics and Gynecology, Damascus University, Damascus, Syria; University Hospital of Obstetrics and Gynecology, Damascus University, Damascus, Syria; Faculty of Medicine, Damascus University, Damascus, Syria; University Hospital of Obstetrics and Gynecology, Damascus University, Damascus, Syria

**Keywords:** postpartum hemorrhage, uterine compression suture, B-lynch suture, uterine atony, placenta accreta spectrum

## Abstract

Uterine compression suture is an alternative choice for managing postpartum hemorrhage and preserving the uterus. However, the required sutures and needles may not be available in developing countries. The 70 mm round-bodied needle was not available in our country. Therefore, we used a 60 mm straight needle mounted to a No. 2/0 Nylon suture and a 40 mm round-bodied needle mounted to a No. 2 Vicryl suture. The free ends were tied effectively with three knots to ensure stability, and the straight needle was used to penetrate the uterus. By using this modification, we took advantage of the length of the straight needle to pass through the uterine muscle at every entry/exit point, which was something that could not be achieved by the small, round-bodied needle (40 mm) available in our hospital. Our modification showed a good prognosis and subsequent successful pregnancy.

## Introduction

Postpartum hemorrhage (PPH) is the primary cause of maternal mortality globally and is linked to more devastating outcomes in developing countries [1, 2]. While hysterectomy can be a life-saving procedure, it may yield mental repercussions and impact the patient’s quality of life, in addition to the loss of reproductive capability [2, 3]. Therefore, to avoid hysterectomy, uterine compression sutures (UCSs) have evolved as an alternative choice for addressing severe and even mild PPH, effectively preserving the uterus in 76%–100% of cases [4, 5].

The B-lynch suture is the most commonly used technique that was first described in 1997 [4]. It requires a long, absorbable (i.e. chromic catgut, Monocryl, or Vicryl) suture mounted to a 70 mm round-bodied hand needle [4, 6]. Many studies modified the procedure as a technique but neglected the impact of using the appropriate needles and sutures on morbidity, clinical course, and future fertility [4, 5]. Furthermore, poverty, war and civilian unrest, and a lack of medical supplies may prevent developing nations from having the necessary sutures and needles [2, 6].

In Syria, the 70 mm round-bodied needle is not available. Therefore, an alternative approach that is effective, affordable, easy to learn, and rapid to perform with the limited, available equipment is required to save lives. Herein, we report the management of two cases of PPH with different underlying causes using a modified B-lynch suture.

This report of the two cases has been reported in line with the SCARE Criteria [7].

## Case 1

A 27-year-old female gravida 4 para 2 in her 29th week arrived at our emergency department with severe vaginal bleeding and abdominal pain for the past 2 h. She stated, “I haven’t been able to feel my baby’s movements since the bleeding.” The most recent of her two cesarean deliveries was two and a half years ago. Otherwise, her past medical and family history was unremarkable. Her blood pressure was 90/60 mmHg, her heart rate was 120 bpm, and her temperature was 37.3°C. She had a rigid abdomen on physical examination. Laboratory tests revealed hemoglobin levels of 10.9 mg/dl with normal kidney function tests and coagulation profile. Transvaginal ultrasound showed no fetal heart activity and confirmed the diagnosis of placental abruption.

She underwent a cesarean section under general anesthesia and delivered a stillbirth fetus. She had a Couvelaire uterus as a complication of placental abruption and subsequently developed severe PPH. Uterine massage and administration of oxytocin and ergometrine failed to stop the bleeding, and we decided to apply B-lynch sutures as a final attempt to stop the bleeding.

**Figure 1:**
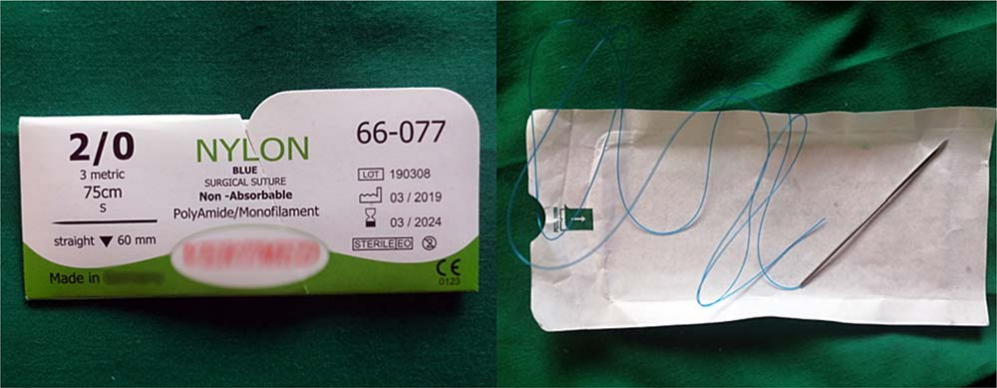
Shows the 60 mm straight needle mounted to the Nylon suture (both commercial name and country of manufacturing were blurred).

**Figure 2:**
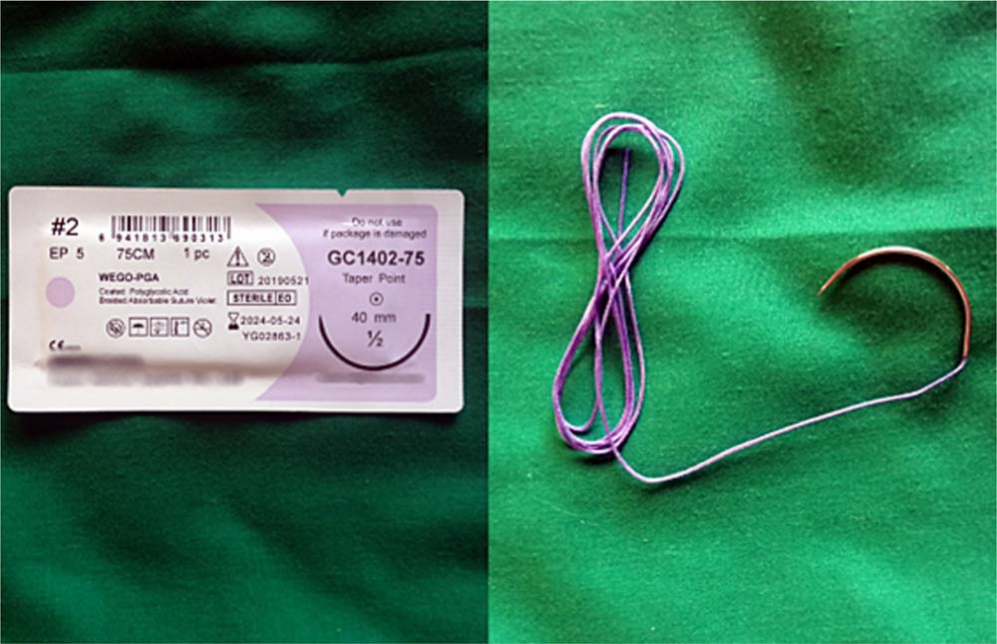
Shows the 40 mm round-bodied needle mounted to the Vicryl suture (both commercial name and country of manufacturing were blurred).

**Figure 3:**
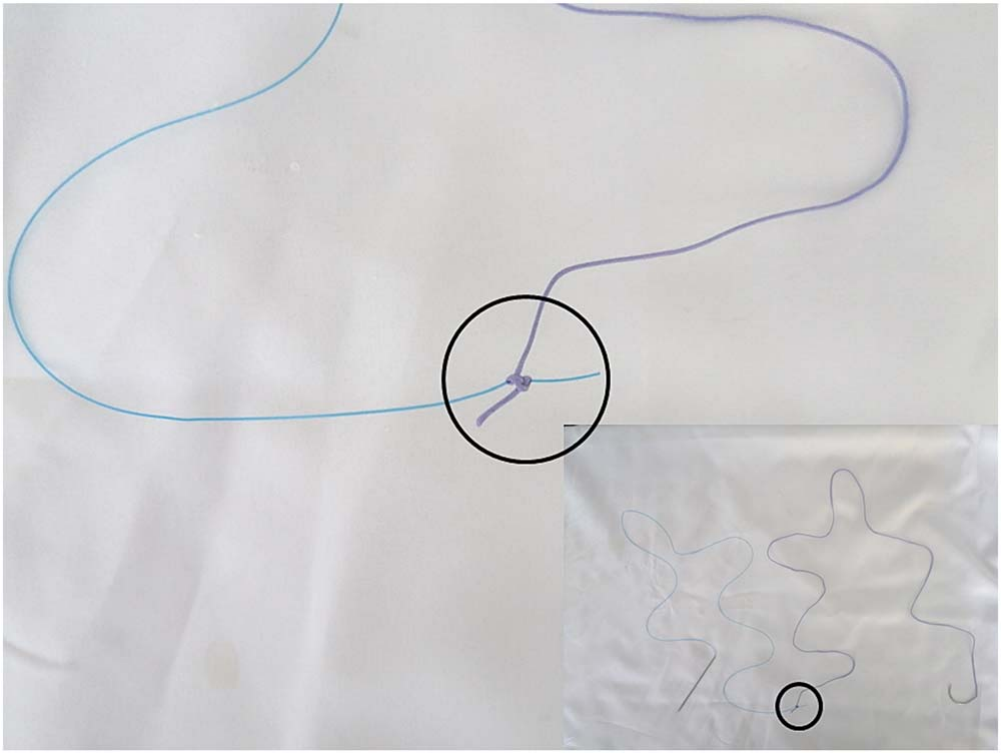
Illustrates how the free ends were tied (marked by the black circle).

**Figure 4:**
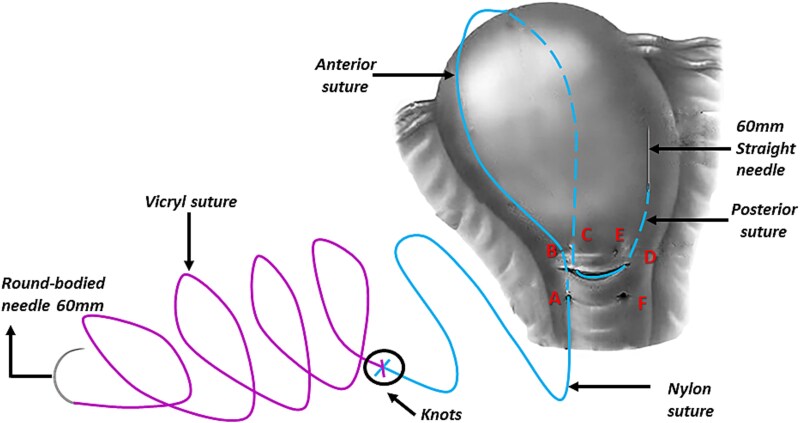
Illustrates how we penetrated the uterus with the straight needle that was pulling the Nylon and the Vicryl sutures (this figure is our own).

**Figure 5:**
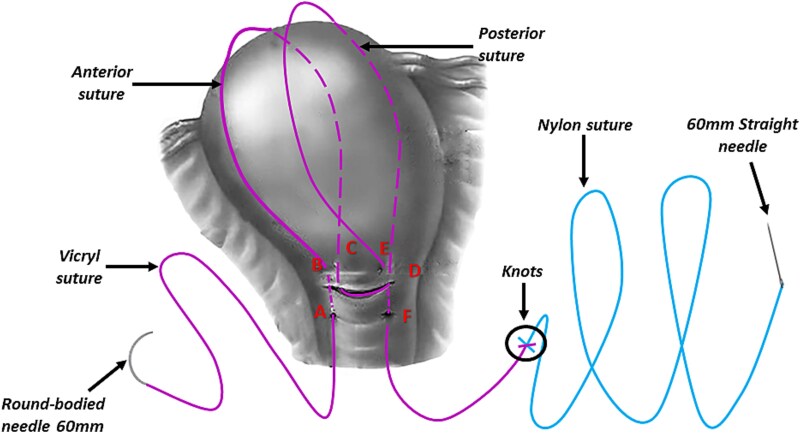
Illustrates the final step of the procedure and how the straight needle and the Nylon suture are out of the uterus and that only the Vicryl suture embraces the uterus (this figure is our own).

**Figure 6:**
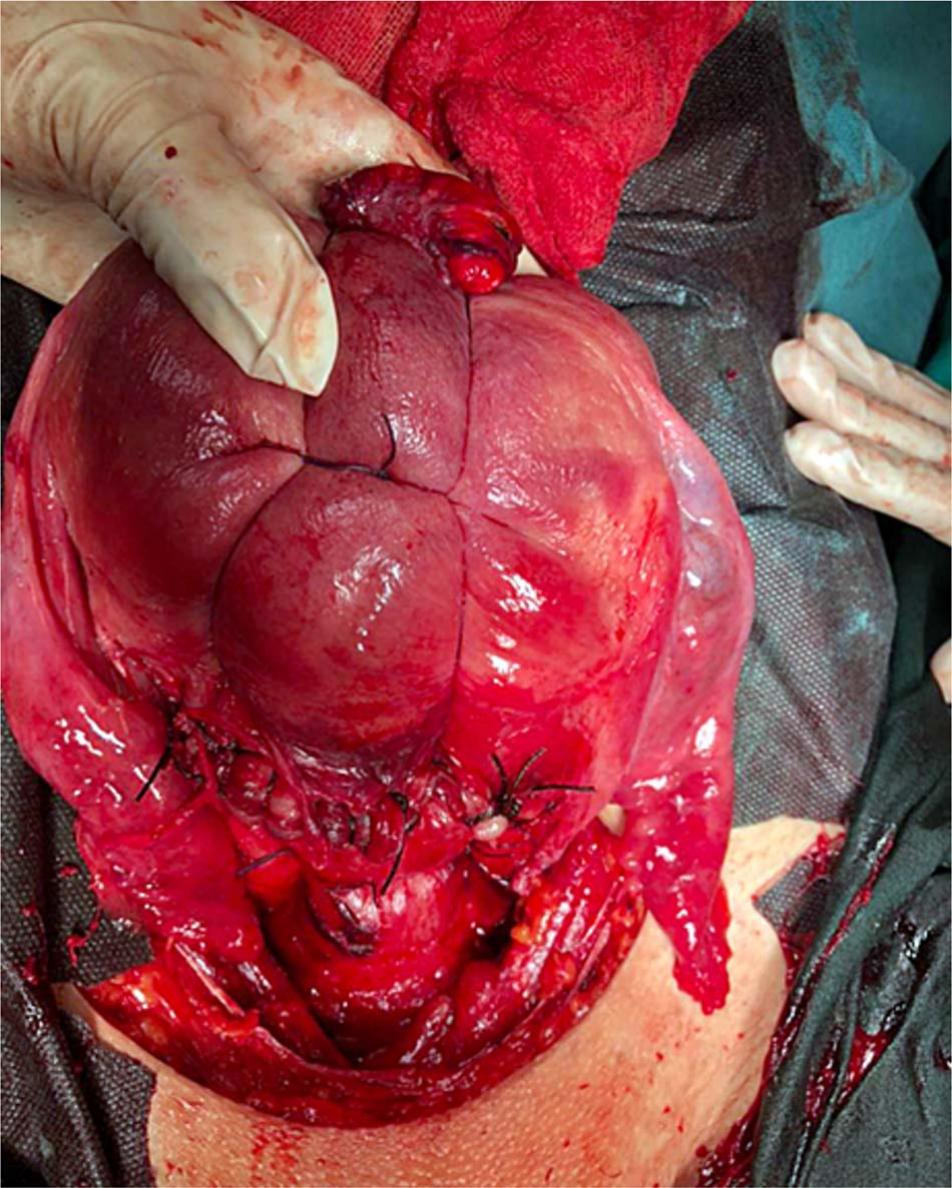
B-lynch suture after it has been applied.

However, the 70 mm round-bodied needle was not available in our hospital. Therefore, we used a 60 mm straight needle mounted to a No. 2/0 Nylon suture (75 cm in length) (Fig. 1) and a 40 mm round-bodied needle mounted to a No. 2 Vicryl suture (75 cm in length) (Fig. 2). The free ends were tied effectively with three knots to ensure stability (Fig. 3). It is worth mentioning that this knot would not cause any minor trauma to the uterus. We penetrated the uterus with the straight needle 3 cm below the incision line (Fig. 4, point A). The needle was interspersed through the uterine cavity and pulled at 3 cm above the incision line (Fig. 4, point B). Then it was vertically looped over the fundus and entered the uterine cavity posteriorly at the same level as the upper anterior entry point (Fig. 4, point C). Then the suture crossed the midline horizontally to exit from the posterior wall (Fig. 4, point D). Then it was looped back over the fundus to the anterior, entered 3 cm above the incision line (Fig. 4, point E), and pulled out 3 cm below the incision line (Fig. 4, point F). Here we want to emphasize that the round-bodied needle was not used and stayed outside the uterus. And, as we were inserting and pulling the straight needle and the Nylon suture, we were pulling the Vicryl suture inevitably, as it was tied to the Nylon one. Before finishing the procedure, we made sure that the straight needle and the Nylon suture were out of the uterus and that only the Vicryl suture embraced the uterus (Fig. 5). Finally, we cut out the Nylon suture and tied the Vicryl suture effectively, which compressed the uterus and succeeded in stopping the bleeding (Fig. 6). We closed the lower segment, followed by all abdominal layers, and transferred the patient to the intensive care unit for observation and blood transfusion.

Using this method, we took advantage of the length of the straight needle (that mounted to the Nylon suture and was tied to the Vicryl suture) to pass through the uterine muscle at every entry/exit point, which was something that could not be achieved by the small, round-bodied needle (40 mm) available in our hospital.

The patient was discharged home after 7 days. She was in good condition and had a follow-up program every 4 weeks. The clinical and ultrasonographic evaluation showed no signs of uterine necrosis or pyemia developed. Seven months later, the patient became pregnant. We ensured a close follow-up throughout pregnancy. A full-term baby was delivered by elective cesarean section without any complications.

## Case 2

A 38-year-old woman, gravida 4 para 3 at 33 weeks gestation, presented to our emergency department with premature labor. She had three previous cesarean deliveries; the last one was 16 months ago. Otherwise, her medical, familial, and surgical history was unremarkable. Ten milligram of Nifedipine three times/day and 6 mg of dexamethasone every 12 h for two consecutive days were administered. She was under close observation and discharged home after a significant improvement.

**Figure 7:**
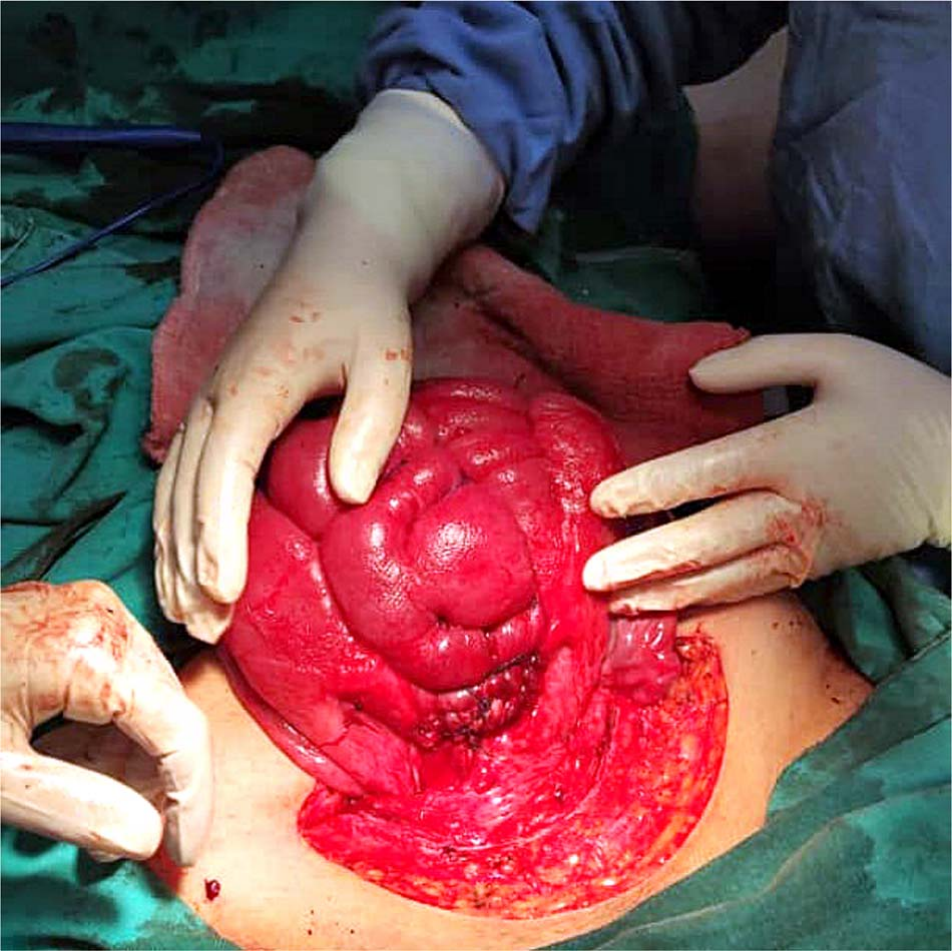
B-lynch suture after it has been applied.

At 36 weeks gestation, she presented with abdominal pain and mild uterine contractions. Her blood pressure was 120/80 mmHg, and her heart rate was 85 bpm, with normal oxygen saturation. Her hemoglobin levels were 11.2 mg/dl, fasting blood glucose was 71 mg/dl. Platelet count, prothrombin time, and partial thromboplastin time were within the reference range. Ultrasound revealed a singleton, live pregnancy, and normal placenta located apart from the lower segment, with uterine scar thickness measuring 2 mm. She underwent a cesarean section under general anesthesia. After the delivery, she experienced uterine atony and severe PPH. Uterine massage failed to stop the bleeding. Intravenous oxytocin and per rectum misoprostol were also administered without any response. Therefore, we decided to perform the same previously described B-lynch suture. Nine horizontal sutures of the modified B-lynch sutures were applied to the body and managed to contract the uterus and stop the bleeding (Fig. 7). Abdominal layers were closed with a drain inside, along with blood transfusion. Five days later, she was doing well and was discharged home with a healthy baby. She had a conventional course of recovery during the follow-up and with no significant findings on the ultrasound.

## Discussion and conclusion

PPH is a life-threatening condition, especially in low-income countries where such cases pose an immense challenge to obstetricians. B-Lynch sutures have been utilized extensively because they are simple, quick, and only require basic surgical skills [2]. However, specialized sutures and needles may not be available in developing countries, such as Syria, where the 70 mm round-bodied needle is not available. To overcome this issue, a 60 mm straight needle mounted to a No. 2/0 Nylon suture (75 cm in length) and a 40 mm round-bodied needle mounted to a No. 2 Vicryl suture (75 cm in length) were used. The free ends were tied effectively with three knots to ensure stability, and the straight needle was used to penetrate the uterus. By using this modification, we took advantage of the length of the straight needle (that mounted to the Nylon suture and was tied to the Vicryl suture) to pass through the uterine muscle at every entry/exit point, which was something that could not be achieved by the small, round-bodied needle (40 mm) available in our hospital.

In the first case, placental abruption, our modification not only successfully preserved the uterus, but it also made it possible for a successful fertilization and delivery to occur within 1 year after the procedure. Our outcome could be supported by several reports which showed that B-lynch suture had no effect on fertility or the results of subsequent pregnancies [2]. In the second case, uterine atony, the modification also succeeded in preserving the uterus; however, more sutures were required. As a result, it is reasonable to expect that the more serious the hemorrhage, the more sutures will be needed to raise the tension and compression force and stop the bleeding.

Many studies have discussed the efficacy of different sizes of sutures and needle types, making the procedure more applicable. A systematic review of the differences among suture types stated that Catgut or Polyglactin 910 are the most commonly used sutures [8]. It also concluded that the transfusion rate in UCSs was significantly lower when using No. 1 rather than No. 2. However, uterine preservation rates were similar between the same groups.

Yano *et al.* [9] used a long, 100 mm needle with a relatively large radius (42.5 mm) to control PPH following the removal of placenta accreta. This needle easily penetrated the uterine wall and achieved hemostasis. Another study described the efficacy and safety of using a Catgut round needle (No. 2 70 mm) on 13 patients who presented with severe PPH due to uterine atony. This technique stopped the bleeding in 12 patients with a success rate of 92.2%. Only one patient had persistent bleeding after applying the sutures, and a subsequent hysterectomy was required [10]. Another study used No. 1 chromic catgut with a large, curved, round needle “the Meydanli compression suture” to manage PPH due to uterine atony in seven patients [11]. This technique stopped the bleeding in six patients and did not in one patient with placenta increta who needed a subsequent hysterectomy to stop the bleeding.

In summary, these two examples do not support the idea that this new change is better than others. Even more encouraging evidence for this change in our circumstances, however, comes from the favorable prognosis and the following successful pregnancy outcomes. Furthermore, we propose that the more sutures needed to enhance the tension and compression force and halt the bleeding, the more severe the hemorrhage. Finally, more trails are required to make a consensus regarding this modification.

## Data Availability

The datasets used and/or analyzed during the current study are available from the corresponding author on reasonable request.
